# Drug Repurposing Approaches for the Treatment of Influenza Viral Infection: Reviving Old Drugs to Fight Against a Long-Lived Enemy

**DOI:** 10.3389/fimmu.2019.00531

**Published:** 2019-03-19

**Authors:** Andrés Pizzorno, Blandine Padey, Olivier Terrier, Manuel Rosa-Calatrava

**Affiliations:** Virologie et Pathologie Humaine–VirPath Team, Centre International de Recherche en Infectiologie (CIRI), INSERM U1111, CNRS UMR5308, ENS Lyon, Université Claude Bernard Lyon 1, Université de Lyon, Lyon, France

**Keywords:** influenza virus, antivirals, antiviral resistance, drug repurposing, drug repositioning, drug discovery, drug combination, transcriptional profiling

## Abstract

Influenza viruses still constitute a real public health problem today. To cope with the emergence of new circulating strains, but also the emergence of resistant strains to classic antivirals, it is necessary to develop new antiviral approaches. This review summarizes the state-of-the-art of current antiviral options against influenza infection, with a particular focus on the recent advances of anti-influenza drug repurposing strategies and their potential therapeutic, regulatory and economic benefits. The review will illustrate the multiple ways to reposition molecules for the treatment of influenza, from adventitious discovery to *in silico*-based screening. These novel antiviral molecules, many of which targeting the host cell, in combination with conventional antiviral agents targeting the virus, will ideally enter the clinics and reinforce the therapeutic arsenal to combat influenza virus infections.

## Influenza Viruses, a Long-Lived Threat for Populations

“*A piece of bad news wrapped up in a protein*,” definition of a virus by Sir Peter Medawar.

Despite its apparent blandness for the collective mindset of an important portion of the society, the intrinsic morbidity and mortality as well as the related deaths because of bacterial superinfections or exacerbation of chronic illnesses, make of influenza infections a major and recurrent global public health concern. Indeed, human influenza type A and B viruses are responsible for annual flu epidemics marked by up to 1 billion infections, 3–5 million severe cases and 300,000–650,000 deaths worldwide, with an huge economic burden in terms of medical visits, hospitalizations, work/school absenteeism. and productivity loss (1–3). As members of the *Orthomyxoviridae* family, influenza viruses (type A, B, C, or D) are enveloped viruses harboring a negative-sense single-stranded RNA segmented genome. In such segmented nature of the viral genome resides the capacity of influenza viruses to form new reassortant strains following the concomitant infection of a host with more than one strain of human, and/or animal origin, a phenomenon so far observed only among type A influenza viruses [reviewed in (4)]. Owing to viral reassortment, the genetic baggage of progeny viruses does not exactly match that of one of the “parental” strains but a combination of both. Depending on the specific combination of genetic segments, and notably in the case of a human influenza strain acquiring the Hemagglutinin (HA) and/or Neuraminidase (NA) major surface antigens from animal origin, reassortment events can result in an *antigenic shift*, defined as the generation of a new virus with antigenic properties drastically different from those of the circulating strains. Should this new variant be sufficiently antigenically different to escape the repertoire of pre-existing immunity in the population, it might rapidly disseminate and replace the circulating strains, hence triggering a global influenza pandemic. Although relatively rare–three veritable pandemics occurred during the 20th Century and one so far in the twenty-first century–the outbreak of pandemics is a quite unpredictable event that might entail potentially devastating effects [reviewed in (5)], particularly considering the contemporary state of affairs regarding global transportation and trade, migration, and the narrowing interface between rural and overcrowded urban areas.

Influenza vaccination constitutes the most effective strategy to prevent seasonal flu and its clinical complications, mainly among high-risk populations such as very young children, the elderly, pregnant women, immunocompromised patients as well as people with obesity, diabetes, or cardiorespiratory comorbidities (6, 7). Nevertheless, current flu vaccination still presents several limitations that make it fall short of expectations in terms of effectiveness. The short duration of vaccine-induced immunity coupled with the intrinsic *antigenic drift* of influenza viruses resulting from the gradual accumulation of point mutations in the antigenic sites of the HA (and to a lesser extent the NA) surface protein underscore the need of the annual reformulation of vaccine composition. Moreover, the length of the current vaccine manufacturing process (at least 6 months to produce sufficiently large vaccine quantities) demands continual strain selection to be done approximately 8 months before the next flu season (6, 8). Should an antigenic drift occur during this time window, the possibility of a mismatch between the vaccine composition and circulating strains might negatively affect protection. Even in the absence of seasonal mismatches or the emergence of pandemic strains, insufficient vaccine coverage and suboptimal uptake in specific target groups (i.e., the elderly or the immunocompromised) also compromise vaccine effectiveness. Furthermore, despite the recent progress made in the pursue of the “Holy Grail” of a universal influenza vaccine that can provide broader, long-lasting protection against both matching, and antigenically diverse influenza strains (9, 10), their clinical effectiveness remains to be evaluated, hence highlighting the need of complementary therapeutic approaches to manage influenza infections.

Besides vaccination, antiviral drugs represent the other pillar for the control of seasonal influenza epidemics and play a central role as major prophylactic and therapeutic agents in the event of a pandemic outbreak. In that regard, this review summarizes the state-of-the-art of current antiviral options against influenza infection, with a particular focus on the recent advances of anti-influenza drug repurposing strategies and their potential therapeutic, regulatory and economic benefits. This review presents examples of the multiple ways to reposition molecules for the treatment of influenza, from adventitious discovery to *in silico*-based screening. These novel antiviral candidates, many of which target the host cell, could also be used in combination with conventional virus-targeted antiviral agents in order to reinforce our very limited therapeutic arsenal against influenza virus infections.

## Current Antiviral Options for Treating Influenza Infections

As mentioned above, antivirals are key players in pandemic preparedness programs, being the first choice for the treatment of infected patients as well as for preventive post-exposure prophylaxis of those potentially exposed to the new virus, especially during the initial pandemic period in which no vaccine is available. Antivirals are as well important in the normal seasonal setting. Although their use is mostly focused on the treatment of severely ill patients and the immunocompromised, some countries, including the USA and Japan, regularly resort to antivirals for the management of uncomplicated influenza in otherwise healthy patients (11, 12). To date, only two classes of antiviral agents are globally approved and available for the treatment of influenza infections: M2 ion-channel blockers and neuraminidase (NA) inhibitors. The first class includes adamantane derivatives, amantadine and rimantadine, which inhibit proton conductivity of the M2 ion channel of influenza A viruses hence preventing the viral uncoating step of the viral replication cycle. Nevertheless, although quite efficient in their early days, widespread dissemination of the S31N (and to a much lesser extent V27A) M2 resistance mutation in post-2006 H3N2 and post-2009 H1N1 circulating strains prompted the WHO to remove both amantadine and rimantadine from the list of recommended anti-influenza agents for clinical use, in 2009 (6). As a result, NA inhibitors stand as the only influenza antivirals currently recommended by the WHO (13).

NA inhibitors are competitive analogs of sialic acid, the preferred influenza receptor on the host cell's surface. By binding to the broadly conserved active site of the NA, NA inhibitors interfere with the sialidase enzymatic activity of the viral protein, which is essential for the release of newly formed progeny viruses from the infected cell, hence preventing the spread of infection to the rest of the respiratory tissue (14). Three NA inhibitors are currently licensed worldwide for the treatment of influenza A and B infections: oseltamivir, zanamivir, and peramivir. Oral oseltamivir (administered as its prodrug oseltamivir phosphate) is the most largely used of the three, whereas inhaled zanamivir is not recommended for very young children nor for individuals with underlying respiratory conditions, and intravenous peramivir is prioritized in hospitalized patients that cannot receive oral treatment (15). Additionally, inhaled laninamivir, a single-dose long lasting NA inhibitor, is approved in Japan for the prevention and treatment of influenza A and B in both adult and pediatric patients (16). It is important to note that some degree of skepticism is still present regarding the real efficacy of NA inhibitors, notably following the 2014's Cochrane clinical meta-analysis that reported only a minimal shortening of influenza symptoms in children and adults with uncomplicated influenza but not in hospitalized patients (17). Nevertheless, actual evidence-based consensus points to a moderate efficacy of NA inhibitor treatment in reducing symptom duration, pneumonia, hospitalization and mortality, especially when administered within 48 h from symptom onset (18, 19). Conversely, delayed treatment initiation is associated with compromised efficacy but may yet be beneficial in at-risk patients. Moreover, the emergence of NA inhibitor-resistant virus variants is a matter of concern, with particularly higher frequencies among children and the immunocompromised (20). The H275Y NA substitution is the main mutation responsible for both oseltamivir and peramivir resistance in H1N1 viruses while R292K and E119V are the most commonly reported in H3N2 viruses, these latter two also conferring reduced susceptibility to zanamivir and laninamivir (17, 21). Even if nowadays the prevalence of drug-resistance in circulating strains is quite low (≤1%), evidence form pre-2009 seasonal strains has proved that, given the appropriate conditions, resistance could rapidly disseminate to attain a prevalence of 90–100% (17, 21). In that regard, the relatively recent detection of localized clusters of NA inhibitor-resistant H1N1pdm09 viruses harboring the H274Y mutation combined or not with I222R/V NA substitutions (22, 23) strengthens the importance of continuous surveillance.

In addition to M2 ion channel blockers and NA inhibitors, two small molecules that target the viral RNA-dependent RNA polymerase, favipiravir and baloxavir marboxil, are undergoing clinical evaluation in the US and Europe but already obtained approval by Japanese Health authorities. Favipiravir is a nucleoside analog that acts as a competitive inhibitor of viral polymerase substrate, approved since 2014 for the treatment of influenza infections with newly emerging strains and/or resistant to other antiviral agents. However, despite the apparent high threshold for drug resistance (24) and broad-spectrum antiviral potential notably validated in the context of recent Ebola virus outbreaks (25), recent results of Phase II/III randomized trials on its therapeutic efficacy against uncomplicated influenza were not completely conclusive (26). Baloxavir marboxil is a selective inhibitor of the cap-dependent endonuclease activity of the influenza viral PA polymerase subunit (27), therefore interfering with the cap-snatching activity of the viral polymerase complex. In that regard, a very recent report disclosed for the first time the results of two randomized (Phases II and III) clinical trials evaluating the efficacy of a single-dose oral treatment with baloxavir marboxil in otherwise healthy outpatients with acute uncomplicated influenza, compared with placebo and a regular 5-day treatment with oseltamivir (28). Overall, baloxavir marboxil and oseltamivir moderately reduced the time to symptom alleviation compared to placebo, while the former outperformed the two others in reducing viral loads. These results prompted the US Food and Drug Administration (FDA) to approve Xofluza® (baloxavir marboxil) for the treatment of acute uncomplicated influenza in patients 12 years of age and older who have been symptomatic for no more than 48 h (29). Nevertheless, this first antiviral flu treatment with a novel mechanism of action approved by the FDA in nearly 20 years does not seem to escape the problem of all other virus-targeted anti-influenza agents. The emergence of virus variants (mostly due to the I38T/M PA amino acid substitutions) conferring significant levels of reduced susceptibility to baloxavir marboxil was observed in up to 9.7% of the patients receiving the drug (28, 30).

Overall, Table 1 summarizes the main characteristics of the abovementioned currently available antiviral options for influenza. Such limited therapeutic arsenal coupled with the recurrent risk of emerging drug-resistance highlights the obvious unmet need of novel approaches to complement existing therapies with new anti-influenza drugs.

**Table 1:** Currently approved drugs for the treatment of influenza viral infections.

**International non-proprietary name**	**Pharmaceutical brand names (examples)**	**Antiviral class**	**Antiviral activity**	**Clinical indication**	**Resistance reported**	**Discovery/Reference**
Amantadine hydrochloride	Mantadix Symmetrel Symadine Osmolex ER	M2 ion-channel blockers	Blocks influenza virus uncoating and entry into host cell	High risk old adults and children Prophylaxis Or treatment 24/48 post symptoms appearance	YES	1963 (31)
Rimantadine hydrochloride	Roflual Flumandine					1969 (32)
Oseltamivir phosphate	Tamiflu	NA inhibitors	Sialic acid structural analog, competitive inhibitor of the influenza viral neuraminidase substrate	Children, adolescent and adults 48 h from symptom onset	YES	1998 (33)
Zanamivir	Relenza			Children and adults ≥5 years (prophylaxis) ≥7 years (treatment) 48 h from symptom onset		1993 (34)
Peramivir	Rapivab Peramiflu Rapiacta			Children, adolescent and adults intravenous peramivir is prioritized in hospitalized patients that cannot receive oral treatment 48 h from symptom onset		2000 (35)
Laninamivir octanoate	Inavir			Children and adults inhaled laninamivir Prevention adults and pediatric patients		2000 (36)
Favipiravir	Avigan	Polymerase inhibitor	Nucleoside analog, competitive inhibitor of viral RNA-dependent RNA polymerase substrate	Limited to cases in which other influenza antiviral drugs are ineffective or not sufficiently effective	YES	2002 (37)
Baloxavir marboxil	Xofluza		Selective inhibitor of the cap-dependent endonuclease activity of the influenza viral PA polymerase subunit	Treatment of acute uncomplicated influenza in patients 12 years of age and older who have been symptomatic for no more than 48 h		2018 (27)

## What is Drug Repurposing?

“*The most fruitful basis for the discovery of a new drug is to start with an old drug,”* famously stated the 1998 Nobel Prize in Physiology and Medicine Laureate, Sir James Black.

Despite the enormous scientific and technological advances that the field of biomedical research has witnessed in the last 20–30 years, this scenario failed to efficiently translate into significant improvement on the success rate of the classic “from the bench to the bedside” target-centered, mechanistically biased *de novo* drug discovery process (38). Indeed, with an almost unchanged total number of 25–30 novel molecules out of the approximately 50 new drugs yearly approved by the FDA (39), biopharmaceutical experts estimate that only 12% of drug candidates that make it into Phase I clinical trials receive the final green light (40). In other words, of 5,000–10,000 compounds that come from classic drug discovery, only one is likely to be approved. The causes of this phenomenon are multifactorial, including the targeting of more intricate diseases, limitations of reductionist experimental models to reproduce biological complexity, increased regulatory stringency, tolerability issues, and unexpected side effects. Altogether, the total R&D process leading to the introduction of a new drug in the market demands on average 13–15 years and between U$S 1.5 and 2.6 billion (40–42).

**Figure 1:**
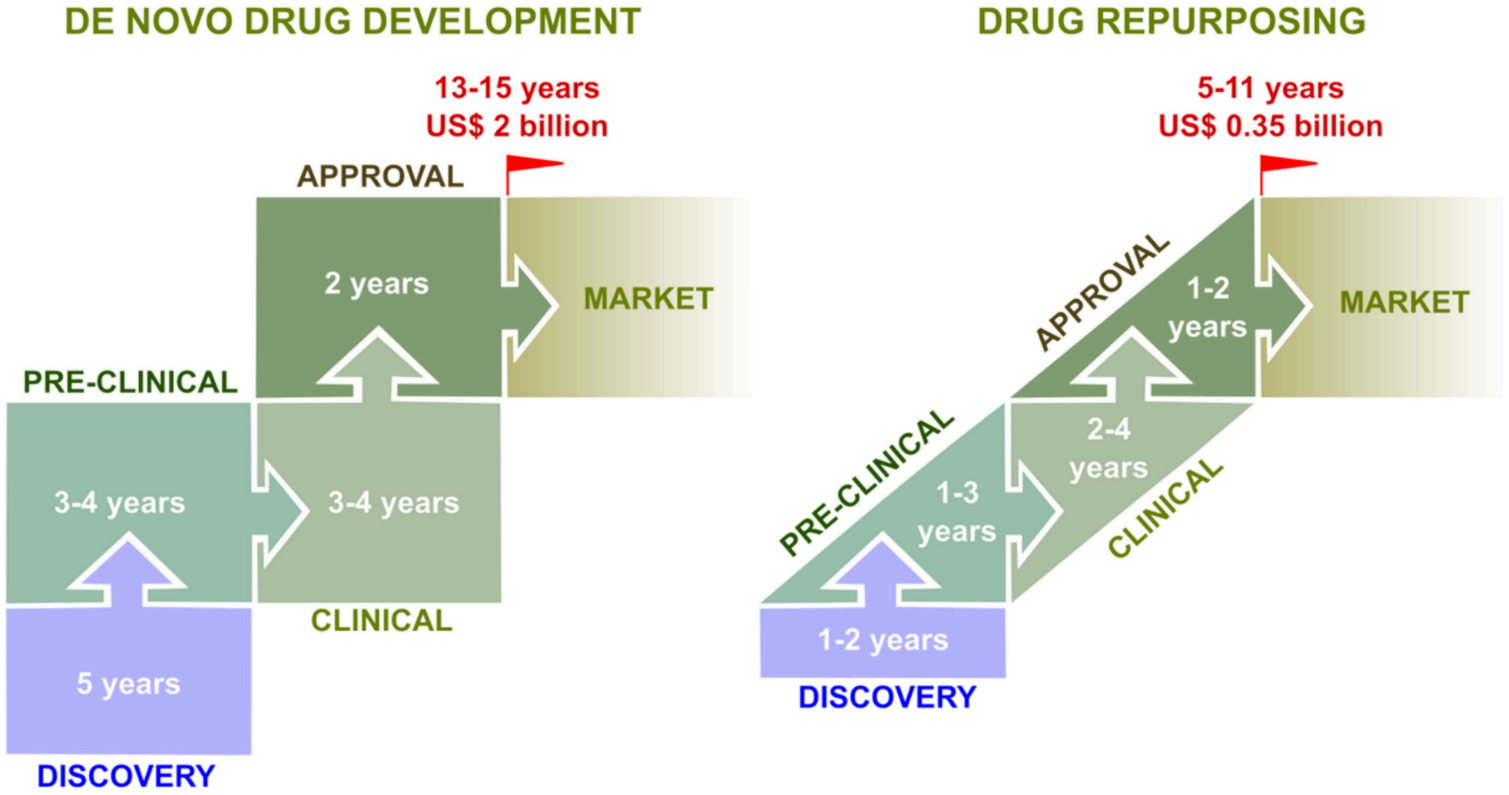
From the bench to the bedside: comparison between *de novo* drug development and drug repurposing. *De novo* (classic) drug development constitutes a time-consuming and expensive process. From initial discovery to market, it generally takes 13–15 years and costs up to US$ 2 billion, with a very low success rate (10%). In contrast, drug repurposing approaches offer several advantages. Indeed, the time frame from discovery to market is shorter (5–11 years), less expensive (US$ 350 million), and with a higher success rate (30%), mostly because a large part of preclinical and clinical testings (e.g., safety, formulation, posology) have been already performed for the drug's initial therapeutic indication (41, 42).

In this context, drug repurposing stands as a worthwhile attractive alternative to fill part of this so-called innovation gap. Drug repurposing, also termed drug repositioning, defines the process of identifying and validating a new therapeutic indication for an existing or developmental drug (38, 42, 43). The basis of drug repurposing relies on bypassing long, risky and expensive preclinical and early clinical evaluation stages by focusing on available extensive human clinical, pharmacokinetics and safety data as the starting point for further development (Figure 1). An extended definition could also include not only already marketed drugs but also “sleeping” candidates that have seen their development abandoned in advanced phases of clinical evaluation (e.g., Phase II/III trials) due to non-satisfactory efficacy for their first intended medical use, which might find a second life in a novel therapeutic indication Noteworthy, repurposing arguably accounts for 30% of the new drug products approved by the FDA (44).

In practice, the concept of drug repurposing represents a broad term encompassing many different, though not mutually exclusive, experimental approaches to recognize potential new applications outside the scope of the original medical indication (42), including:

### Serendipitous Observations

Some of the best-known success stories of drug repurposing have their starting point on serendipitous observations recorded in the context of either preclinical models of disease or pre-/post-approval clinical trials, leading to a subsequent rationalized evaluation and validation of the new treatment potential (41). Thalidomide and sildenafil are two examples of such key observations. The first one was initially introduced as an anti-nausea for pregnant women but had to be rapidly removed from the market due to its teratogenicity. Further research enabled this molecule as well as some derivatives to be repurposed for the treatment of leprosy and multiple myeloma (45). Sildenafil, on the other hand, never reached the market for its originally intended use in the treatment of hypertension but the observed side-effects on erectile dysfunction ended in its approval in under the commercial name of Viagra®. More recently, sildenafil found a third life under the commercial brand of Revatio® for the treatment of pulmonary hypertension (46).

### Target-Based Repurposing

Although serendipitous observation has historically proved its usefulness, the intrinsic necessity of the casual observation of an unintended and usually infrequent second benefit poses a significant hurdle for exploiting the full potential of drug repurposing, for which more controlled, systematic methodologies are needed. Target-based repurposing relies on having previous knowledge of the specific molecular or cellular determinant/function target recognized by the drug intended to be repurposed. If new research finds out that target is plays an important role in a condition or disease other than the original indication, there is a potential for repurposing. Of note, the target might but not necessarily has to play the same role in both conditions. For example, in the case of the previously mentioned favipiravir, the drug plays the same role as viral RNA polymerase inhibitor against both influenza and Ebola viruses. On the other hand, the Abelson tyrosine-protein kinase 2 (Abl2), target of the anticancer drug imatinib, has been found to be required for efficient fusion and release of severe acute respiratory syndrome coronavirus (SARS-CoV) and Middle East respiratory syndrome coronavirus (MERS-CoV) pseudovirions into the cytoplasm of the infected cell, a key step for viral replication (47).

An alternative scenario of target-based repurposing can happen when a particular drug of known mechanism of action is found to have a new molecular/cellular target, and this previously unrecognized second target is associated with a different disease. The molecule is therefore said to present *polypharmacology*-related features, meaning the capacity to act on multiple targets (48, 49). Polypharmacological phenomena includes both a single drug acting on multiple targets of a unique disease pathway, or a single drug acting on multiple targets pertaining to multiple disease pathways (50). In fact, polypharmacology is usually responsible for treatment toxicity or other undesirable adverse events, but some of these “side-effects” might also lead to drug repurposing, as further exemplified in the next sections. During the last decade, an increasing number of studies converged on proposing that many drugs, initially designed for a unique therapeutics target, are in fact expected to hit on average between 6 and 13 different targets (51, 52).

### Phenotypic Screening

One major limitation of the target-based drug repurposing model relies on its dependence on the existing scientific knowledge of the drug/disease mechanism(s) of action/pathology as well as on potential alternative targets, which is usually incomplete. In other words, we cannot fully anticipate the repurposing potential of a drug unless we have characterized its molecular/cellular target(s), or if we do not know that a given drug target plays an important role on a particular disease. Phenotypic screening of bioactive molecule libraries in different experimental cell-based or *in vivo* disease models without the need of *a priori* knowledge or consideration of the target and/or mechanism of action the candidate was designed to modulate can provide valuable contribution to overcome this constraint (53). Indeed, despite this approach has been questioned due to the fact that the expected altered phenotype readout as a surrogate of an exploitable biological effect induced by the drug candidate might account for an important number of false positive “hits,” it is nonetheless true that the contribution of high-throughput phenotypic screening to first-in-class small molecule drug discovery exceeded that of target-based approaches (54, 55). In that regard, many well-annotated collections of small-molecule libraries could be readily made available through different collaborative and/or commercial partnerships in order to accelerate drug repurposing through hypothesis biased or unbiased phenotypic screening [reviewed in (54–57)].

### *In silico*-Assisted Repurposing

With the advent of big data and systems biology, computer-based approaches are gaining increasing acceptance in the field of drug discovery, and drug repurposing is not an exception. Besides the inclusion of constantly emerging “omics” (e.g., transcriptomic, proteomic, metabolomic) data to expand our current knowledge of drug/disease-associated mechanisms, *in silico* data mining and modeling tools have pushed our capacity to analyze data to the next level (58–60). These *in silico* methods include the screening of chemical, biological, and text databases, analysis of quantitative structure-activity relationships, pharmacophores, homology models, and other molecular modeling approaches as well as network analysis of biological functions, machine learning and almost any other analysis tools that include using a computer (61–64). In that regard, proper mining of biological, chemical and clinical datasets, has proved effective in unveiling novel relationships (65, 66). Moreover, another level of complexity can be added by combining, for example, epidemiologic information obtained in-house and/or from publicly available literature databases with *in vitro* experimental molecule screenings with the aim to identify novel indications, as in the case of digoxin and prostate cancer (67, 68). Indeed, the real power of computer-assisted drug repurposing resides on adopting an integrative strategy that combines the predictive and analytic capacity of *in silico* tools with some of the target biased or unbiased experimental evaluation/validation methods previously mentioned. This “systems pharmacology” approach (69–71) across the boundaries of traditional disciplines would put researchers in a better-informed position to design more comprehensive repurposing strategies with more effective predictive capacity and, hopefully, improved candidate success rates.

## The Emergence of Drug Repurposing Approaches in the Field of Antiviral Drug Discovery

These last 10 years, there has been a remarkable growing interest for drug repurposing in the field of antiviral drug discovery, fueled by the incontestable reality of many known viral infections still lacking specific treatment. This interest is inversely correlated with the very low number of classic antiviral molecules that have been market-approved these last 5 years, mostly for the treatment of hepatitis C virus or HIV-related pathologies (72). The best example of antiviral drug repurposing approaches are emerging viruses such as Ebola, Zika virus or MERS-CoV, for which there is an urgent and cost-effective need for therapeutics solutions. Indeed, to rapidly propose a solution in the context of a viral outbreak, one interesting approach consists to look at the available pharmacopeia used to treat pathogens. For example, chloroquine, a major antimalarial drug, has been proposed for the treatment of filoviral infections, and more largely for the treatment of other emerging pathogens, as it targets endosomal acidification, a pivotal step in the replication cycle of a large number of viruses (73, 74). Another interesting illustration is the previously cited example of favipiravir, which proved its repurposing potential for the treatment of Zika or Ebola viral infections (25, 43, 75).

## Drug Repurposing for Influenza Viral Infection

As mentioned before, the intrinsic ever-evolving nature of the virus, high transmissibility, host promiscuity, suboptimal vaccine efficacy, limited antiviral arsenal, and zoonotic, and pandemic potential are more than convincing factors to consider influenza viruses as attractive targets for drug repurposing. Despite many interesting omics-based approaches (76) or high-throughput screening of specific drug libraries, such as kinase inhibitors (77), no anti-influenza agent issued from drug repurposing has yet reached regulatory market approval. However, advances made during the last years forecast optimism. The following selected examples constitute a very good illustration of the diversity and capabilities of drug repurposing strategies for influenza infection. An exhaustive list of anti-influenza candidates issued from drug repurposing approaches is presented in Table 2.

**Table 2:** Drug repurposing approaches for the treatment of influenza viral infections.

**Name**	**Initial indication**	**Initial activity**	**Repurposing approach**	**Anti-influenza activity**	**Status**	**References**
Statins (i.e., Atorvastatin)	Cholesterol modulators	HMG-CoA reductase inhibitor	Serendipity	Immunomodulator	Phase II (NCT02056340)	(78–80)
Nitazoxanide	Anti-parasitic Chronic hepatitis	Inhibition of the pyruvate: ferredoxin/flavodoxin oxidoreductase cycle		HA maturation & transport inhibition	Phase III (NCT03336619)	(81, 82)
PPAR antagonists (i.e., Gemfibrozil)	Anti-hyperlipidemic	Hepatic glucogenesis inhibitor	Serendipity & Phenotypic screening	Immunomodulator	Preclinical	(83, 84)
LASAG (BAY81-87981)	Anti-inflammatory	NF-kB inhibitor	Serendipity & Target-based	NF-kB inhibition	Phase II (2012-004072-19)	(85, 86)
Celecoxib	Anti-inflammatory	COX-2 inhibitor	Target-based	Immunomodulator	Phase III (NCT02108366)	(87, 88)
Etanercept	Anti-inflammatory Rheumatoid arthritis	Anti-TNF-α agent		Immunomodulator	Preclinical	(89)
Metformin	Approved Type 2 diabetes drug	Hepatic glucogenesis inhibitor	Phenotypic screening	Immunomodulator Autophagy induction	Preclinical	(78, 83)
Gemcitabine	Approved anti-cancer drug	Ribonucleotide reductase inhibitor		Immunomodulator	Preclinical	(90)
Dapivirine	Phase III anti-HIV drug	Reverse transcriptase inhibitor		vRNP transport inhibition	Preclinical	(91)
Trametinib	Approved anti-cancer drug	MEK1/2 inhibitor		vRNP transport inhibition	Preclinical	(92)
Lisinopril	Approved anti-hypertensive drug	peptidyl dipeptidase inhibitor	*In-silico* assisted, target-based screening	NA inhibitor	Preclinical	(93)
Naproxen	Approved NSAID Phase I anticancer	COX-2 inhibitor		NP-RNA binding inhibitor	Phase II (ISRCTN11273879)	(94)
Nalidixic acid	Approved antibiotic	Bacterial DNA replication inhibitor		NA inhibitor	Preclinical	(95)
Dorzolamide	Approved anti-glaucoma drug	Carbonic anhydrase inhibitor		NA inhibitor	Preclinical	
Ruxolitinib	Approved for myelofibrosis treatment	JAK inhibitor		Virion formation & vRNA incorporation inhibition	Preclinical	(96)
Midodrine	Approved anti-hypotensive drug	Adrenergic alpha agonist	*In-silico* assisted, phenotypic screening	Immunomodulator ?	Phase II (NCT01546506)	(97)
Diltiazem	Approved anti-hypertensive drug	Ca2+ channel inhibitor		Immunomodulator ?	Phase II (NCT03212716)	(98)

The case of statins is arguably the best-known example of anti-influenza repurposing issued from clinical observations. In the early 2000s, clinicians observed that besides the cardioprotective activity of statins, these hydroxyl methylglutaryl-coenzyme A (HMG-CoA) reductase inhibitors approved for their use as cholesterol metabolism regulators could have pleiotropic anti-inflammatory and immunomodulatory effects, which could be of benefit to improve survival of patients with severe influenza (78–80). Although many mouse and observational studies account for the protective role of statins in pneumonia, most *in vivo* studies reported so far failed to clearly demonstrate such a beneficial effect in the specific context of influenza infection (99–102). On the other hand, a few but not all observational studies highlighted an association between statin treatment with up to 41% reduction of 30-day all-cause mortality in patients hospitalized with laboratory-confirmed seasonal influenza (103–105). A randomized placebo-controlled Phase II clinical trial (NCT02056340) aimed at evaluating the potential effect of atorvastatin to reduce the severity of illness in influenza-infected patients is currently undergoing.

Nitazoxanide is another illustration of a serendipitous repurposing approach, and probably one of the most promising examples. Nitazoxanide is a thiazolide anti-infective initially licensed for the treatment of parasitic infections, for which anti-influenza properties were first documented by Rossignol et al. (81). Interestingly, the proposed mode of action of nitazoxanide toward influenza is clearly distinct to that for which it was designed in its initial indication, acting at the post-translational level by selectively blocking the maturation of the viral glycoprotein HA, with a consecutive impact on its intracellular trafficking and insertion into the host plasma membrane (81, 106). This drug presents potent antiviral activity against a large panel of circulating strains (82). The effectiveness of nitazoxanide in treating patients with non-complicated influenza was successful in a Phase IIb/III trial (107) and is currently being assessed in a Phase III clinical trial (NCT01610245).

BAY81-8781/LASAG (D, L-Lysine acetylsalicylate-glycine), a modified version of the anti-inflammatory drug acetylsalicylic acid (ASA) licensed for intravenous and inhalation delivery, is currently investigated as an anti-influenza treatment as a result of a mixed serendipitous and target-based repurposing strategy. It was initially shown that ASA had interesting antiviral effects against influenza viruses *in vitro* and *in vivo* via the inhibition of the NF-kB activating kinase IkkB, which negatively impacts influenza vRNP transport and release of infectious viral particles (108–110). However, due to the pharmacokinetic limitations of ASA, the LASAG modified version with improved stability and tolerability was developed. Like ASA, this molecule also demonstrates antiviral activity against several human and avian influenza viruses *in vitro*. In a mouse infection model, inhalation of LASAG resulted in reduced lung viral titers and protection of mice from lethal infection (85). More recently, a Phase II proof-of-concept study comparing LASAG *versus* placebo in patients with severe influenza (all patients receiving Tamiflu as standard of care treatment) demonstrated that aerosolized LASAG improved the time to symptom alleviation compared to placebo, despite the absence of a statistically significant reduction of viral load in LASAG-treated group (86).

Naproxen constitutes a nice example of *in-silico* & target-based strategy for the identification of new antivirals. Lejal et al. used a structure-based modeling approach to identify drugs of interest directed against the nucleoprotein (NP) of influenza A virus, using the X-ray structure of the RNA-free NP of H1N1 as prototype. An *in-silico* screening, focused of a defined specific site of NP structure, has identified naproxen, a known inhibitor of inducible cyclooxygenase type 2 (COX-2) commonly used as non-steroidal anti-inflammatory drug. This identified molecule has shown antiviral properties against influenza A virus *in vitro* and *in vivo* (94). More recently, naproxen analogs with improved efficacy have been developed, showing high level of inhibition of both NP-RNA and NP-polymerase subunit PA complexes, without parallel inhibition of COX-2 (111, 112). Interestingly, in contrast to other examples of drug repurposing strategies, the example of naproxen remains virus-targeted and future works will determine if this drug will present the same Achille's heel than classic antivirals regarding selection of antiviral resistance.

The last two examples of this chapter are midodrine and diltiazem, that we identified as influenza antivirals in the context of an *in-silico* assisted strategy based on transcriptional profiling. An emerging approach in drug repurposing is based on signature matching, which consists of comparing a specific characteristic of a drug–its cellular signature–to that of a disease (42). This approach, mostly based on transcriptomic data, was successfully exploited to identify drug repurposing opportunities in a large range of therapeutics areas, and notably in the field of oncology and rare diseases (42). Our group was the first to transpose this approach to the field of viral infectious diseases, thanks to the development and democratization of DNA-microarray and more recently RNAseq techniques. In a proof-of-concept study using an *in vitro* model of infection, we postulated that host global gene expression profiling can be considered as a “fingerprint” or signature of any specific cell state, including during infection or drug treatment, and hypothesized that the screening of databases for compounds that counteract virogenomic signatures could enable rapid identification of effective antivirals (97). Among the molecules identified *in silico*, midodrine, an adrenergic alpha receptor agonist widely used to treat hypotension, demonstrated very interesting *in vitro* antiviral activities (97). These results prompted the Phase II clinical evaluation of midodrine (NCT01546506) for the treatment of uncomplicated seasonal flu in primary care centers.

Based on this previous proof-of-concept obtained from *in vitro* gene expression profiles, we further improved the strategy by analyzing upper respiratory tract clinical samples collected from a cohort of influenza A(H1N1)pdm09-infected patients and determined their respective transcriptomic signatures. We then performed an *in-silico* drug screening and identified a list of candidate bioactive molecules with signatures anti-correlated with those of the patient's acute infection state. The potential antiviral properties of selected market-approved molecules were firstly validated *in vitro*, and the most effective compounds were further compared to oseltamivir for the treatment of influenza A(H1N1)pdm09 virus infections in mice and in a physiological *in vitro* model of reconstituted human airway epithelia (MucilAir™). These results notably highlighted diltiazem, a calcium channel blocker used as an anti-hypertensive drug, as a very promising repurposed host-targeted inhibitor of influenza infection (98). An ongoing French multicenter randomized clinical trial is investigating the effect of diltiazem-oseltamivir bitherapy compared with standard oseltamivir monotherapy for the treatment of severe influenza infections in intensive care units (FLUNEXT trial NCT03212716).

## Virus-Targeted vs. Host-Targeted Therapy, Why Not Both?

“*Two are better than one, because they have a good return for their labor*” Ecclesiastes 4:9-10.

The concept of antiviral combination therapy was originally pioneered for antiretroviral treatments, with the primary goal of preventing or at least delaying the emergence of drug resistance via the targeting of multiple steps of the viral cycle (113). Another expected complementary goal is to obtain additive or synergistic effects by combining drugs, a “double-trigger” effect, to increase effectiveness and/or reduce dosage. In the context of influenza infections, the combination of classic antivirals, mostly NA inhibitors, was explored by several research groups, including ours, with relatively mixed conclusions. For example, in a mouse model, the combination of oseltamivir with zanamivir was shown to be not superior to zanamivir monotherapy in the context of influenza A(H3N2) or A(H1N1)pdm09 infection (114). A clinical trial was conducted during the A(H1N1)pdm09 pandemic in 2009-2010 (COMBINA trial NCT00830323) and failed to demonstrate whether oseltamivir/zanamivir combination therapy improved or reduced the effectiveness of oseltamivir alone in the treatment of influenza infections in community patients (115). Other clinical investigations have shown a greater effectiveness of such combination therapy to reduce influenza transmissibility (116).

As most alternative antiviral strategies for the treatment of influenza infections, including those related to drug repurposing and targeting the host instead of viral determinants, an emerging trend consists to propose innovative therapies that combine classic antivirals with host-targeting drugs, which starts to show promising results (87). For example, Belardo et al. have demonstrated, in cell culture-based assays using different human and avian models, that the combination of NA inhibitors and nitazoxanide presents synergistic anti-influenza effects (117). Convincing results were also obtained using a combination treatment including naproxen. In a clinical trial enrolling hospitalized patients infected by influenza A(H3N2), combination therapy with naproxen, oseltamivir, and clarithromycin showed improved efficacy in terms of hospital stay duration and patient mortality, when compared to oseltamivir treatment alone (118). In the context of the evaluation of the antiviral activity of diltiazem in the reconstituted human airway epithelium model MucilAir™, our group demonstrated that the diltiazem-oseltamivir combination treatment conferred a greater reduction of apical viral titers than that was measured with the same-dose monotherapy, with a marked delay of viral production (98). An ongoing French multicenter randomized clinical trial is investigating the effect of diltiazem-oseltamivir bitherapy compared with standard oseltamivir monotherapy for the treatment of severe influenza infections in intensive care units (FLUNEXT trial NCT03212716).

Altogether, these results plead in favor of the use of drug repurposing for the improvement of the current standard of care anti-influenza therapy. In contrast to other technological domains, the innovation is not necessary chasing and replacing the established standard, and future works are still necessary to investigate the real impact of these novel “host & virus-targeted” multi-therapy approaches on the management and control of the emergence of viral resistance.

## Concluding Remarks

“*We do not need to find new drugs; rather we need to find the patients who can benefit from existing drugs”* the saying goes. Although somehow exaggerated, this statement summarizes pretty clearly the essence behind the drug repurposing initiative. Finding new indications for already-existing drugs has many benefits, mainly by improving cost-effectiveness, reducing risks, and shortening time to market (37, 41). The purpose of this review was to foster discussion on drug repurposing as an option to complete and implement our current anti-influenza therapeutic arsenal. We are facing an important need for the development of novel antiviral strategies that improve treatment effectiveness–especially in the case of severe diseases–and that are less prone to selection for antiviral resistance. In that regard, the identification and validation by different and complementary means of repurposed drugs is incontestably of great interest, notably in combination with current classic virus-targeted inhibitors. In addition, the deposition of data, including negative results, into public database should be encouraged, as it would facilitate efforts to repurpose licensed or orphaned drugs, and consecutively increase our chances to find new efficient antiviral drugs. With a growing number of academic groups and pharmaceutical companies working on this emerging field, we should most certainly see interesting progress and efficient novel anti-influenza therapies reaching regulatory market approval in a near future.

In the context of a globalized world facing major vicissitudes including population dynamics, climate change and the multiple emergence/re-emergence of zoonotic viruses, the effectiveness and reaction force of the classic *de novo* development of antivirals is challenged. Despite inherent limits, drug repurposing offers a very large palette of possibilities to rapidly and efficiently find new antiviral drugs.

## Author Contributions

All authors listed have made a substantial, direct and intellectual contribution to the work, and approved it for publication.

### Conflict of Interest Statement

AP, BP, OT, and MR-C are co-inventors of two patent applications filed by INSERM, Université Claude Bernard Lyon 1, Laval University and Hospices Civils de Lyon for the repurposing of drugs as anti-influenza agents (WO/2016/146836 and WO/2017/174593).
